# Synthesis and crystal structure of 2-[(2,3,5,6-tetra­fluoro­pyridin-4-yl)amino]­ethyl methacrylate

**DOI:** 10.1107/S2056989023004334

**Published:** 2023-05-26

**Authors:** Shayla M. J. Overstreet, Ian M. Genzia, Alexander Nguyen, Zachary J. Auleciems, Abby R. Jennings, Andrew J. Peloquin

**Affiliations:** a Department of Chemistry, United States Air Force Academy, Colorado Springs, CO 80840, USA; University of Aberdeen, United Kingdom

**Keywords:** crystal structure, perfluoro­pyridine, C—H⋯F inter­action

## Abstract

In the title compound, C_11_H_10_F_4_N_2_O_2_, the conformation about the N—C—C—O bond is *gauche* [torsion angle = 61.84 (13)°]. In the crystal, N—H⋯O hydrogen bonds link the mol­ecules into [010] chains, which are cross-linked by C—H⋯F and C—H⋯π contacts. Hirshfeld surface analysis was conducted to aid in the visualization of these various influences on the packing. This analysis showed that the largest contribution to the surface contacts arises from F⋯H/H⋯F inter­actions (35.6%), followed by O⋯H/H⋯O (17.8%) and H⋯H (12.7%).

## Abstract 2

In the crystal structure of the title compound, the packing is driven by C—H⋯F, N—H⋯O and C—H⋯π contacts. Hirshfeld surface analysis showed that the largest contribution to the surface contacts arise from F⋯H/H⋯F inter­actions.

## 1. Chemical context

Perfluoro­pyridine (C_5_NF_5_; PFPy) is an ideal candidate to use in the preparation of complex fluorinated compounds and materials as PFPy is reactive towards nucleophilic addition (Sandford, 2012 ▸). Furthermore, our group and others have demonstrated that this addition can be regio-selectively controlled, with stoichiometric addition to the 4-(*para-*) position being exclusive with a broad range of nucleophiles (Brittain & Cobb, 2019 ▸; Peloquin *et al.*, 2020 ▸; Seyb & Kerres, 2013 ▸). Sequential addition can also be accomplished at the 3,5-(*meta-*) positions (Corley *et al.*, 2019 ▸; Houck *et al.*, 2021 ▸). As part of our ongoing work in this area, the synthesis and single-crystal structure of the title compound, C_11_H_10_F_4_N_2_O_2_, is reported herein. 
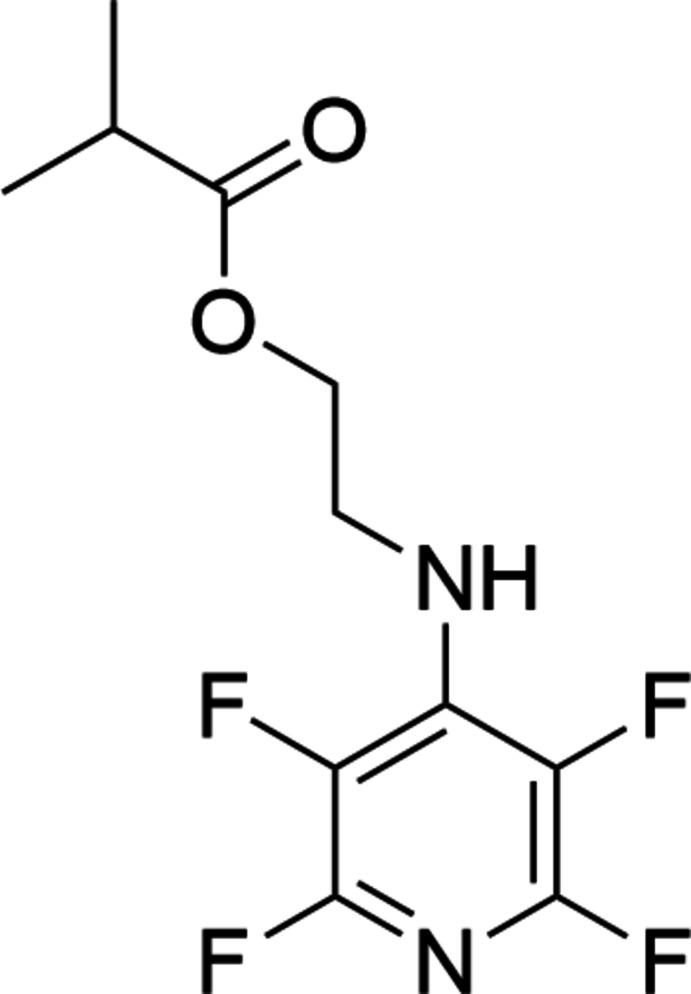


## 2. Structural commentary

**Figure 1:**
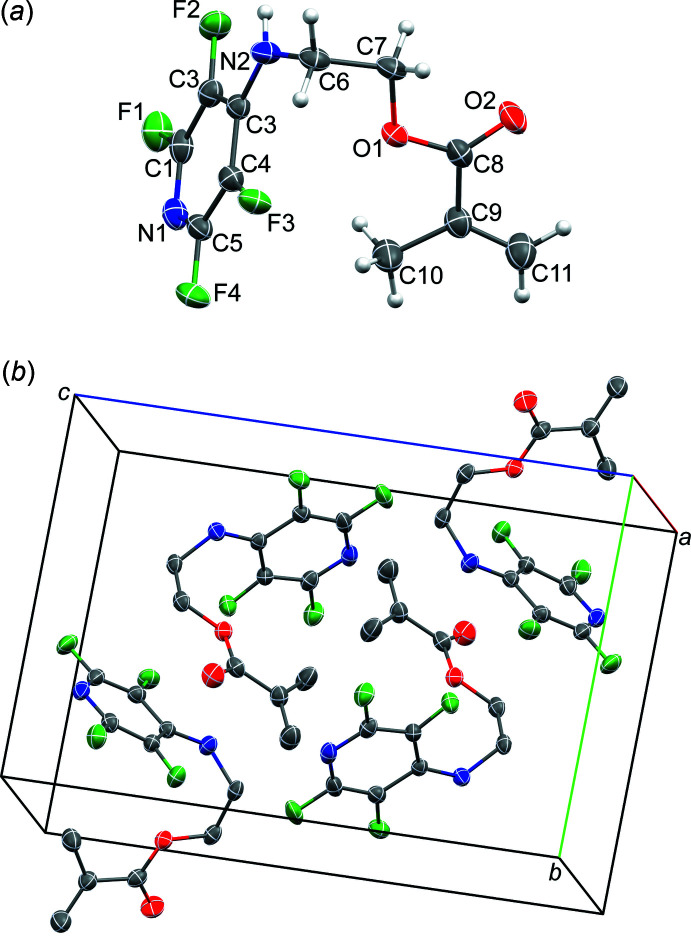
The mol­ecular structure of the title compound (*a*) and the unit-cell packing (*b*). Displacement ellipsoids are shown at the 50% probability level.

The title compound (Fig. 1 ▸) crystallizes in the monoclinic space group *P*2_1_/*n* with one mol­ecule in the asymmetric unit. The N2—C6 bond is rotated by only 15.81 (8)° from the C1–C5/N1 ring plane, presumably to encourage conjugation of the nitro­gen atom lone pair with the aromatic ring π system, which is reflected in the C3—N2 bond length of 1.3522 (16) Å; the C3—N2—C6—C7 torsion angle is −81.68 (16)°. The amine nitro­gen atom (N2) and ester oxygen atom (O1) are *gauche* to one another, with N2—C6—C7—O1 = 61.84 (13)°. The C10 methyl group is oriented in such a fashion as to enable a weak C—H⋯π inter­action with the aromatic ring of an adjacent mol­ecule (Table 1 ▸).

## 3. Supra­molecular features

The main directional inter­actions in the crystal structure of the title compound are of the type C—H⋯F, N—H⋯O and C—H⋯π (Table 1 ▸). The N—H⋯O hydrogen bonds link the mol­ecules into [010] chains, with adjacent mol­ecules related by a 2_1_ screw axis. Weak hydrogen-bonding inter­actions are observed between one hydrogen atom bound to each carbon atom of the two-carbon (C6/C7) linker unit between the amine nitro­gen atom and the ester, and F3 as acceptor. One of these inter­actions is intra­molecular (C6—H6*A*⋯F3) with the other being inter­molecular (C7—H7*A*⋯F3). A hydrogen-bonding inter­action occurs between the secondary amine and the carbonyl oxygen atom (N2—H1*N*2⋯O2). Finally, a weak C—H⋯π inter­action is observed between H7*B* and the pyridine ring π system.

**Figure 2:**
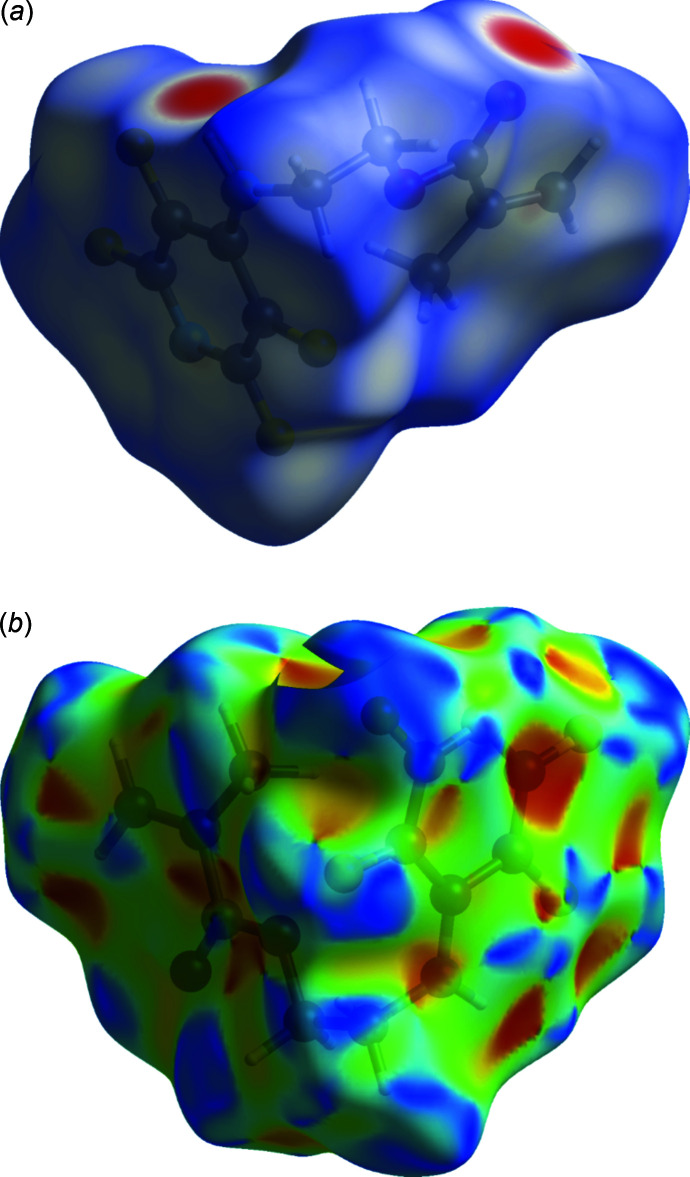
Map of *d*_norm_ (*a*) and shape index (*b*) onto the Hirshfeld surface for the title compound.

Hirshfeld surface analysis was used to investigate the presence of hydrogen bonds and inter­molecular inter­actions in the crystal structure. The Hirshfeld surface analysis (Spackman & Jayatilaka, 2009 ▸) and the associated two-dimensional fingerprint plots (Spackman & McKinnon, 2002 ▸) were generated by *CrystalExplorer17.5* (Turner *et al.*, 2017 ▸), using standard surface resolution with the three-dimensional *d*_norm_ surfaces plotted over a fixed color scale of −0.025 (red) to 1.38 (blue) a.u.; the pale-red spots symbolize short contacts and negative *d*_norm_ values on the corresponding surface plots shown in Fig. 2 ▸, associated with their relative contributions to the Hirshfeld surface.

**Figure 3:**
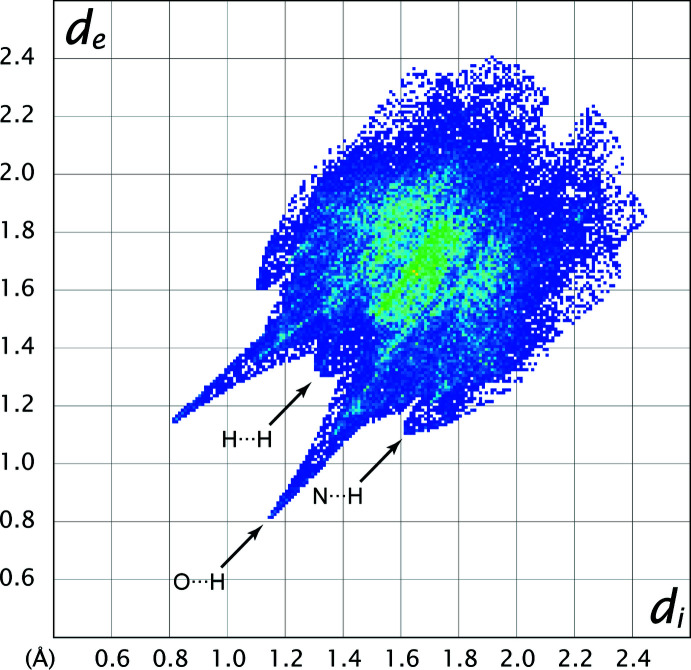
The overall two-dimensional fingerprint plot for the title compound.

The largest contribution to the overall crystal packing is from F⋯H/H⋯F inter­actions (35.6%) (Table 2 ▸). However, the spike in the fingerprint plot (Fig. 3 ▸) associated with this contact is masked by that of the O⋯H/H⋯O contacts, which appears in the plot at 0.80 Å < (*d_i_* + *d_e_*) < 1.15 Å. The H⋯H and C⋯H/H⋯C contacts have the second and third largest contributions, at 17.8 and 12.7%, respectively. Smaller spikes on the shoulder of the fingerprint plot, appearing at 1.15 Å < (*d_i_* + *d_e_*) < 1.160 Å, correspond to the N⋯H/H⋯N contacts.

## 4. Database survey

A search of the November 2019 release of the Cambridge Structure Database (Groom *et al.*, 2016 ▸), with updates through November 2012, was performed using the program *ConQuest* (Bruno *et al.*, 2002 ▸). The search was limited to 2,3,5,6-tetra­fluoro­pyridine-based compounds with a secondary amine nitro­gen atom bound to the ring in the 4-position. This search resulted in 19 hits: the C—C—N—C torsion angles indicate planarity, presumably due to conjugation of the nitro­gen atom lone pair into the pyridine ring π system, in the majority of cases. In cases of non-planarity, this is typically due to steric factors of the substituent on the nitro­gen atom or conjugation of that nitro­gen lone pair into the π system of the substituent. For example, in CSD refcode NIXMEN (Ranjbar-Karimi *et al.*, 2008 ▸), the bulk of a phenyl ring attached to the nitro­gen atom subsitutent discourages planarity, resulting in a torsion angle of 37.4°. In TAPRAD (Yamaguchi *et al.*, 1992 ▸), the conjugation of the nitro­gen lone pair is into a urea substituent, *vice* the pyridine ring, with a torsion angle of 38.7°.

## 5. Synthesis and crystallization

2-[(Perfluoro­pyridin-4-yl)amino]­ethan-1-ol was synthesized using a known method and used without further purification (Peloquin, *et al.*, 2020 ▸). Methacryloyl chloride was purchased from Sigma and distilled under reduced pressure prior to use.

A 500 ml round-bottom flask equipped with an addition funnel was charged with 2-[(perfluoro­pyridin-4-yl)amino]­ethan-1-ol (13.4 g, 62.3 mmol), tri­methyl­amine (10.7 ml, 77.2 mmol) and diethyl ether (300 ml). The solution was stirred under nitro­gen at 273–278 K for 15 minutes. Next, a solution of methacrylol chloride (7.50 ml, 76.8 mmol) in ether (10 ml) was added dropwise to the round-bottom flask using an addition funnel. The solution was allowed to gradually warm to room temperature and was stirred for 96 h under nitro­gen. Precipitated salts were removed by vacuum filtration and the filtrate was concentrated under reduced pressure. Crystals of the title compound in the form of colorless needles were obtained by recrystallization from a solution in warm (∼328 K) hexa­nes (9.0 g, 50.7%): m.p. 335–336 K; ^1^H NMR (500 MHz, CDCl_3_): δ 6.01 (*s*, vinyl, 1H), 5.61 (*s*, vinyl, 1H), 4.90 (*bs*, –N*H*CH_2_CH_2_O–, 1H), 4.38 (*t*, –NHCH_2_C*H_2_* O–, 2H, ^3^ *J* = 5.0 Hz), 3.86 (*q*, –NHC*H_2_* CH_2_O–, 2H, ^3^ *J* = 5.3 Hz), 1.93 (*s*, CH_2_=C(C*H_3_*)–, 3H); ^19^F NMR (471 MHz, CDCl_3_): δ −93.6 (*bs*, 2F), −163.4 (*bs*, 2F); ^13^C NMR (125 MHz, CDCl_3_): δ 167.8 (*C*=O), 135.8 (CH_2_=*C*(CH_3_)-), 126.4 [*C*H_2_=C(CH_3_)–], 63.7 (–O*C*H_2_CH_2_NH–), 44.1 (–OCH_2_ *C*H_2_NH–), 18.3 [CH_2_=C(*C*H_3_)–].

## 6. Refinement

Crystal data, data collection and structure refinement details are summarized in Table 3 ▸. N-bound H atoms were refined freely. C-bound H atoms were positioned geometrically (C—H = 0.95–0.99 Å) and refined as riding with *U*_iso_(H) = 1.2–1.5*U*_eq_(C).

## Supplementary Material

Crystal structure: contains datablock(s) I. DOI: 10.1107/S2056989023004334/hb8066sup1.cif

Structure factors: contains datablock(s) I. DOI: 10.1107/S2056989023004334/hb8066Isup2.hkl

Supporting information file. DOI: 10.1107/S2056989023004334/hb8066Isup3.cml

CCDC reference: 2263932

Additional supporting information:  crystallographic information; 3D view; checkCIF report

## Figures and Tables

**Table 1:** Hydrogen-bond geometry (Å, °) *Cg*11 is the centroid of the C1–C5/N1 ring.

*D*—H⋯*A*	*D*—H	H⋯*A*	*D*⋯*A*	*D*—H⋯*A*
N2—H*N*2⋯O2^i^	0.811 (18)	2.119 (17)	2.8296 (15)	146.3 (15)
C6—H6*B*⋯F3	0.99	2.23	2.9004 (15)	124
C7—H7*B*⋯F3^ii^	0.99	2.57	3.5368 (15)	167
C7—H7*B*⋯F4^ii^	0.99	2.63	3.3646 (16)	131
C7—H7*A*⋯*Cg*1^iii^	0.99	2.89	3.6047 (15)	130

Symmetry codes: (i) 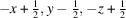 ; (ii) 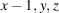 ; (iii) 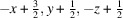 .

**Table 2:** Percentage contribution of inter-atomic contacts to the Hirshfeld surface of the title compound

Contact	Percentage contribution
F⋯H/H⋯F	35.6
H⋯H	17.8
C⋯H/H⋯C	12.7
N⋯H/H⋯N	8.7
O⋯H/H⋯O	8.7
F⋯F	6.3
O⋯C/C⋯O	2.5
F⋯O/O⋯F	2.4
F⋯N/N⋯F	1.8
C⋯F/F⋯C	1.7
C⋯C	1.4
N⋯C/C⋯N	0.3

**Table 3:** Experimental details

Crystal data	
Chemical formula	C_11_H_10_F_4_N_2_O_2_
*M*_r_	278.21
Crystal system, space group	Monoclinic, *P*2_1_/*n*
Temperature (K)	106
*a*, *b*, *c* (Å)	6.8588 (1), 10.7797 (2), 15.7707 (2)
β (°)	91.251 (1)
*V* (Å^3^)	1165.74 (3)
*Z*	4
Radiation type	Mo *K*α
μ (mm^−1^)	0.15
Crystal size (mm)	0.44 × 0.18 × 0.17

Data collection	
Diffractometer	XtaLAB Synergy, Single source at offset/far, HyPix3000
Absorption correction	Gaussian (*CrysAlis PRO*; Rigaku OD, 2019 ▸)
*T*_min_, *T*_max_	0.705, 1.000
No. of measured, independent and observed [*I* > 2σ(*I*)] reflections	33062, 2583, 2211
*R*_int_	0.035
(sin θ/λ)_max_ (Å^−1^)	0.647

Refinement	
*R*[*F* ^2^ > 2σ(*F* ^2^)], *wR*(*F* ^2^), *S*	0.035, 0.090, 1.06
No. of reflections	2583
No. of parameters	177
H-atom treatment	H atoms treated by a mixture of independent and constrained refinement
Δρ_max_, Δρ_min_ (e Å^−3^)	0.28, −0.21

Computer programs: *APEX3* and *SAINT* (Bruker, 2017 ▸), *SHELXT2018/2* (Sheldrick, 2015*a* ▸), *SHELXL2018/3* (Sheldrick, 2015*b* ▸), *Mercury* (Macrae *et al.*, 2020 ▸) and *OLEX2* (Dolomanov *et al.*, 2009 ▸).

## supplementary crystallographic information

### Crystal data

**Table:**

C_11_H_10_F_4_N_2_O_2_	*F*(000) = 568
*M**_r_* = 278.21	*D*_x_ = 1.585 Mg m^−^^3^
Monoclinic, *P*2_1_/*n*	Mo *K*α radiation, λ = 0.71073 Å
*a* = 6.8588 (1) Å	Cell parameters from 19836 reflections
*b* = 10.7797 (2) Å	θ = 2.3–27.2°
*c* = 15.7707 (2) Å	µ = 0.15 mm^−^^1^
β = 91.251 (1)°	*T* = 106 K
*V* = 1165.74 (3) Å^3^	Needle, colourless
*Z* = 4	0.44 × 0.18 × 0.17 mm

### Data collection

**Table:**

XtaLAB Synergy, Single source at offset/far, HyPix3000 diffractometer	2211 reflections with *I* > 2σ(*I*)
Radiation source: micro-focus sealed X-ray tube	*R*_int_ = 0.035
φ and ω scans	θ_max_ = 27.4°, θ_min_ = 2.3°
Absorption correction: gaussian (CrysAlis PRO; Rigaku OD, 2019)	*h* = −8→8
*T*_min_ = 0.705, *T*_max_ = 1.000	*k* = −13→13
33062 measured reflections	*l* = −19→20
2583 independent reflections	

### Refinement

**Table:**

Refinement on *F*^2^	Primary atom site location: dual
Least-squares matrix: full	Hydrogen site location: mixed
*R*[*F*^2^ > 2σ(*F*^2^)] = 0.035	H atoms treated by a mixture of independent and constrained refinement
*wR*(*F*^2^) = 0.090	*w* = 1/[σ^2^(*F*_o_^2^) + (0.0389*P*)^2^ + 0.4834*P*] where *P* = (*F*_o_^2^ + 2*F*_c_^2^)/3
*S* = 1.06	(Δ/σ)_max_ < 0.001
2583 reflections	Δρ_max_ = 0.28 e Å^−^^3^
177 parameters	Δρ_min_ = −0.21 e Å^−^^3^
0 restraints	

### Special details

**Table:**

Geometry. All esds (except the esd in the dihedral angle between two l.s. planes) are estimated using the full covariance matrix. The cell esds are taken into account individually in the estimation of esds in distances, angles and torsion angles; correlations between esds in cell parameters are only used when they are defined by crystal symmetry. An approximate (isotropic) treatment of cell esds is used for estimating esds involving l.s. planes.

### Fractional atomic coordinates and isotropic or equivalent isotropic displacement parameters (Å^2^)

**Table:**

	*x*	*y*	*z*	*U*_iso_*/*U*_eq_	
F1	0.76605 (14)	0.04660 (8)	0.49959 (5)	0.0400 (2)	
F2	0.49913 (12)	0.06997 (8)	0.37165 (5)	0.0334 (2)	
F3	0.92779 (11)	0.36294 (8)	0.24890 (5)	0.0301 (2)	
F4	1.17024 (11)	0.32149 (9)	0.37827 (5)	0.0364 (2)	
O1	0.53522 (13)	0.47979 (9)	0.28749 (6)	0.0261 (2)	
O2	0.30721 (15)	0.62510 (10)	0.30633 (7)	0.0391 (3)	
N1	0.96939 (17)	0.18455 (11)	0.43992 (7)	0.0296 (3)	
N2	0.56174 (17)	0.22514 (11)	0.23936 (7)	0.0267 (3)	
C1	0.8023 (2)	0.12386 (13)	0.43516 (8)	0.0293 (3)	
C2	0.66735 (19)	0.13523 (12)	0.37096 (8)	0.0258 (3)	
C3	0.69653 (18)	0.21724 (12)	0.30299 (8)	0.0228 (3)	
C4	0.87510 (19)	0.28051 (12)	0.30859 (8)	0.0241 (3)	
C5	0.99991 (19)	0.25973 (13)	0.37622 (8)	0.0274 (3)	
C6	0.53726 (19)	0.32687 (13)	0.17900 (8)	0.0266 (3)	
H6A	0.468848	0.295683	0.127349	0.032*	
H6B	0.667341	0.356903	0.162348	0.032*	
C7	0.42353 (19)	0.43344 (13)	0.21475 (8)	0.0272 (3)	
H7A	0.406113	0.499531	0.171663	0.033*	
H7B	0.293237	0.405086	0.232375	0.033*	
C8	0.45919 (19)	0.57648 (13)	0.32828 (9)	0.0276 (3)	
C9	0.5847 (2)	0.61826 (14)	0.40162 (9)	0.0306 (3)	
C10	0.7559 (2)	0.54178 (16)	0.42728 (10)	0.0417 (4)	
H10A	0.843816	0.534069	0.379396	0.063*	
H10B	0.711927	0.459166	0.444381	0.063*	
H10C	0.825125	0.581580	0.475012	0.063*	
C11	0.5387 (3)	0.72537 (16)	0.43920 (10)	0.0430 (4)	
H11A	0.617410	0.756041	0.484859	0.052*	
H11B	0.427248	0.770700	0.420212	0.052*	
HN2	0.467 (3)	0.1814 (16)	0.2450 (10)	0.032 (4)*	

### Atomic displacement parameters (Å^2^)

**Table:**

	*U*^11^	*U*^22^	*U*^33^	*U*^12^	*U*^13^	*U*^23^
F1	0.0534 (6)	0.0383 (5)	0.0284 (4)	0.0099 (4)	0.0041 (4)	0.0129 (4)
F2	0.0342 (5)	0.0303 (4)	0.0360 (5)	−0.0056 (3)	0.0052 (3)	0.0048 (3)
F3	0.0244 (4)	0.0381 (5)	0.0277 (4)	−0.0070 (3)	−0.0027 (3)	0.0070 (3)
F4	0.0247 (4)	0.0491 (5)	0.0351 (5)	−0.0030 (4)	−0.0091 (3)	−0.0017 (4)
O1	0.0213 (5)	0.0304 (5)	0.0264 (5)	0.0017 (4)	−0.0040 (4)	0.0011 (4)
O2	0.0270 (5)	0.0447 (6)	0.0453 (6)	0.0115 (5)	−0.0054 (4)	0.0008 (5)
N1	0.0328 (6)	0.0332 (6)	0.0228 (6)	0.0102 (5)	−0.0038 (5)	−0.0007 (5)
N2	0.0236 (6)	0.0292 (6)	0.0270 (6)	−0.0065 (5)	−0.0051 (4)	0.0039 (5)
C1	0.0382 (8)	0.0281 (7)	0.0216 (6)	0.0111 (6)	0.0035 (5)	0.0028 (5)
C2	0.0276 (7)	0.0241 (6)	0.0260 (6)	0.0012 (5)	0.0038 (5)	−0.0004 (5)
C3	0.0229 (6)	0.0238 (6)	0.0217 (6)	0.0024 (5)	−0.0001 (5)	−0.0018 (5)
C4	0.0242 (6)	0.0263 (6)	0.0217 (6)	0.0012 (5)	0.0001 (5)	0.0008 (5)
C5	0.0228 (6)	0.0329 (7)	0.0265 (7)	0.0043 (5)	−0.0034 (5)	−0.0047 (6)
C6	0.0243 (6)	0.0342 (7)	0.0211 (6)	−0.0043 (5)	−0.0051 (5)	0.0044 (5)
C7	0.0203 (6)	0.0345 (7)	0.0266 (7)	−0.0031 (5)	−0.0062 (5)	0.0062 (6)
C8	0.0227 (6)	0.0309 (7)	0.0295 (7)	0.0034 (5)	0.0021 (5)	0.0065 (6)
C9	0.0310 (7)	0.0341 (7)	0.0269 (7)	0.0055 (6)	0.0005 (5)	0.0016 (6)
C10	0.0419 (9)	0.0459 (9)	0.0369 (8)	0.0100 (7)	−0.0106 (7)	−0.0114 (7)
C11	0.0490 (10)	0.0442 (9)	0.0356 (8)	0.0130 (7)	−0.0057 (7)	−0.0044 (7)

### Geometric parameters (Å, º)

**Table:**

F1—C1	1.3413 (16)	C4—C5	1.3714 (18)
F2—C2	1.3516 (15)	C6—H6A	0.9900
F3—C4	1.3495 (15)	C6—H6B	0.9900
F4—C5	1.3444 (16)	C6—C7	1.505 (2)
O1—C7	1.4540 (15)	C7—H7A	0.9900
O1—C8	1.3367 (17)	C7—H7B	0.9900
O2—C8	1.2105 (16)	C8—C9	1.4961 (19)
N1—C1	1.3201 (19)	C9—C10	1.484 (2)
N1—C5	1.3111 (18)	C9—C11	1.339 (2)
N2—C3	1.3522 (16)	C10—H10A	0.9800
N2—C6	1.4594 (17)	C10—H10B	0.9800
N2—HN2	0.811 (18)	C10—H10C	0.9800
C1—C2	1.3625 (19)	C11—H11A	0.9500
C2—C3	1.4072 (18)	C11—H11B	0.9500
C3—C4	1.4030 (18)		
			
C8—O1—C7	116.31 (10)	C7—C6—H6A	109.1
C5—N1—C1	114.56 (11)	C7—C6—H6B	109.1
C3—N2—C6	126.76 (11)	O1—C7—C6	106.82 (10)
C3—N2—HN2	114.8 (11)	O1—C7—H7A	110.4
C6—N2—HN2	115.4 (12)	O1—C7—H7B	110.4
F1—C1—C2	118.98 (13)	C6—C7—H7A	110.4
N1—C1—F1	116.09 (12)	C6—C7—H7B	110.4
N1—C1—C2	124.93 (13)	H7A—C7—H7B	108.6
F2—C2—C1	120.90 (12)	O1—C8—C9	112.50 (11)
F2—C2—C3	117.95 (11)	O2—C8—O1	122.86 (13)
C1—C2—C3	121.13 (12)	O2—C8—C9	124.62 (13)
N2—C3—C2	119.91 (12)	C10—C9—C8	118.83 (13)
N2—C3—C4	126.72 (12)	C11—C9—C8	117.68 (13)
C4—C3—C2	113.32 (11)	C11—C9—C10	123.46 (14)
F3—C4—C3	121.52 (11)	C9—C10—H10A	109.5
F3—C4—C5	118.55 (12)	C9—C10—H10B	109.5
C5—C4—C3	119.92 (12)	C9—C10—H10C	109.5
F4—C5—C4	117.72 (12)	H10A—C10—H10B	109.5
N1—C5—F4	116.16 (11)	H10A—C10—H10C	109.5
N1—C5—C4	126.12 (13)	H10B—C10—H10C	109.5
N2—C6—H6A	109.1	C9—C11—H11A	120.0
N2—C6—H6B	109.1	C9—C11—H11B	120.0
N2—C6—C7	112.48 (11)	H11A—C11—H11B	120.0
H6A—C6—H6B	107.8		
			
F1—C1—C2—F2	−0.02 (19)	C1—N1—C5—C4	1.0 (2)
F1—C1—C2—C3	178.48 (11)	C1—C2—C3—N2	178.87 (12)
F2—C2—C3—N2	−2.59 (18)	C1—C2—C3—C4	1.25 (18)
F2—C2—C3—C4	179.79 (11)	C2—C3—C4—F3	179.97 (11)
F3—C4—C5—F4	−1.19 (18)	C2—C3—C4—C5	−0.49 (18)
F3—C4—C5—N1	178.86 (12)	C3—N2—C6—C7	−81.68 (16)
O1—C8—C9—C10	−8.07 (19)	C3—C4—C5—F4	179.25 (11)
O1—C8—C9—C11	170.13 (13)	C3—C4—C5—N1	−0.7 (2)
O2—C8—C9—C10	173.30 (15)	C5—N1—C1—F1	−179.66 (11)
O2—C8—C9—C11	−8.5 (2)	C5—N1—C1—C2	−0.2 (2)
N1—C1—C2—F2	−179.47 (12)	C6—N2—C3—C2	160.42 (13)
N1—C1—C2—C3	−1.0 (2)	C6—N2—C3—C4	−22.3 (2)
N2—C3—C4—F3	2.5 (2)	C7—O1—C8—O2	−0.88 (19)
N2—C3—C4—C5	−177.92 (13)	C7—O1—C8—C9	−179.54 (11)
N2—C6—C7—O1	61.78 (13)	C8—O1—C7—C6	179.42 (11)
C1—N1—C5—F4	−178.91 (11)		

### Hydrogen-bond geometry (Å, º)

*Cg*11 is the centroid of the C1–C5/N1 ring.

**Table:**

*D*—H···*A*	*D*—H	H···*A*	*D*···*A*	*D*—H···*A*
N2—H*N*2···O2^i^	0.811 (18)	2.119 (17)	2.8296 (15)	146.3 (15)
C6—H6*B*···F3	0.99	2.23	2.9004 (15)	124
C7—H7*B*···F3^ii^	0.99	2.57	3.5368 (15)	167
C7—H7*B*···F4^ii^	0.99	2.63	3.3646 (16)	131
C7—H7*A*···*Cg*1^iii^	0.99	2.89	3.6047 (15)	130

Symmetry codes: (i) −*x*+1/2, *y*−1/2, −*z*+1/2; (ii) *x*−1, *y*, *z*; (iii) −*x*+3/2, *y*+1/2, −*z*+1/2.
